# Imaging of Non-ossifying Fibromas: A Case Series

**DOI:** 10.7759/cureus.14102

**Published:** 2021-03-25

**Authors:** Jashmitha Rammanohar, Chen Zhang, Azeem Thahir, Matjia Krkovic

**Affiliations:** 1 Medicine, School of Clinical Medicine, University of Cambridge, Cambridge, GBR; 2 Trauma and Orthopaedics, Addenbrooke's Hospital, Cambridge University Hospitals National Health Service Foundation Trust, Cambridge, GBR

**Keywords:** non-ossifying fibroma, bone lesion, paediatric orthopedics

## Abstract

Non-ossifying fibromas (NOFs) are common lesions most frequently found in the growing bones of children and adolescents. Although NOFs are benign, their presence as incidental findings often triggers further investigation as they are sometimes mistaken for more sinister bone lesions such as aneurysmal bone cysts. NOFs can also pose an increased risk of pathological fractures. However, there are currently no guidelines on the follow-up of NOFs. We present a case series of five patients from Addenbrooke’s Hospital with NOFs illustrating their morphological features on plain radiographs and highlighting specific characteristics to support clinicians in diagnosing and managing NOFs.

## Introduction

Non-ossifying fibromas (NOFs) are common bone lesions, estimated to be present in 20-30% of all four to ten-year-olds [1]. They are characterized by benign growths of osseous and fibrous tissue and most frequently affect the metaphysis of the distal femur and the proximal tibia [2]. Although NOFs are benign, their presence on X-ray imaging often leads to further, usually unnecessary investigations such as MRI, CT, and biopsies. It is thought that certain NOFs increase the risk of pathological fractures in the growing bones of children. However, little progress has been made in understanding which of these lesions are at higher risk. The Ritschl stage has been proposed to classify NOFs based on their natural radiological progression. These stages are based on the appearance of NOFs on plain radiographs. Stage A lesions are lucent with clear margins. Stage B lesions are lucent with a thin sclerotic border while stage C lesions show increasing sclerosis and stage D lesions are completely sclerosed [3]. It has been reported that Ritschl stage B lesions are at higher risk of fractures and thus follow-up has been recommended until stage C is achieved [2].

We present five patients found through the Addenbrooke's Hospital's electronic patient record system (EPIC) who were identified with at least one NOF. We identified the common imaging modalities used to investigate these patients and staged these lesions by Ritschl stage, using the methodology implemented by Blaz et al. [4]. The main outcomes analyzed for this case series were (1) the patient's presentation and clinical features, (2) the imaging modalities used and the assessment of NOFs by Ritschl staging on plain radiograph and, (3) assessing the risk of fracture and management of these patients.

## Case presentation

We analyzed patients based on their demographics, clinical presentation, workup, presence of fracture, management, and clinical outcomes. The age at presentation ranged from 9 to 27 years old with three female and two male patients. None of the patients had a concurrent fracture at the site of the NOF or required surgery for the management of their NOF. Plain radiographs of these patients were used to stage their NOFs according to the Ritschl stage. One stage A, one stage B, one stage D, and two stage C lesions at the time of initial presentation were identified after Ritschl staging. These findings are summarised in Table 1.

**Table 1:** Summary of patients' characteristics.

Case Number	Age (yrs)	Gender (M/F)	Greatest Length (cm)	Bone	Location	Ritschl Stage	Imaging Modality at Time of Presentation	Follow-up
1	9	M	3.3	Femur	Diaphysis	D	X-ray and MRI	No
2	15	M	6.7	Tibia	Metaphysis	B	X-ray only	Yes
3	11	F	3.0	Fibula	Diaphysis	A	X-ray and MRI	Yes
4	14	F	2.7	Radius	Metaphysis	C	X-ray and MRI	Yes
5	27	F	4.0	Femur	Metaphysis	C	MRI only	No

Case 1:

A nine-year-old boy presented to the ED with left-sided lower back pain radiating to the groin region. The pain was worse on walking but relieved by sitting and lying down. One café-au-lait spot was noted on the boy’s left arm. Bilateral anteroposterior (AP) and frog-leg lateral radiographs showed no evidence of a slipped upper femoral epiphysis (SUFE) or fracture. Following referral to the pediatric orthopedic clinic, a diagnosis of biomechanical pain was concluded. 

**Figure 1:**
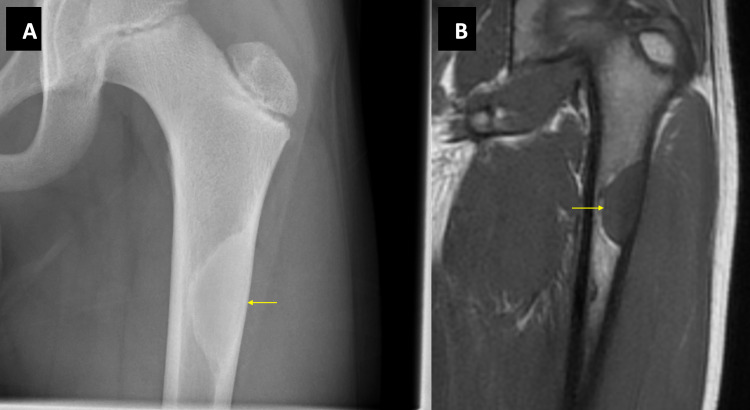
X-ray and MRI images of Case 1. Figure 1A shows a plain radiograph of a completely calcified NOF in keeping with a Ritschl stage D lesion. Figure 1B shows the MRI of the same NOF. NOF: Non-ossifying fibroma.

An incidental finding of a sclerotic lesion of non-aggressive appearance in the left femoral diaphysis was reported on a plain radiograph (Figure 1A). The cortically based lesion measured 3.3 cm in length and had a lentiform shape. At its widest point, the lesion nearly occupied the entire medullary cavity. An MRI scan was requested for further imaging. The T1-weighted MRI showed a discrete sclerotic rim with a distal hyperintense rim (Figure 1B). Despite its thin appearance, the cortex was intact with no periosteal reaction. This was a solitary Ritschl stage D NOF with no concurrent fracture.

**Figure 2:**
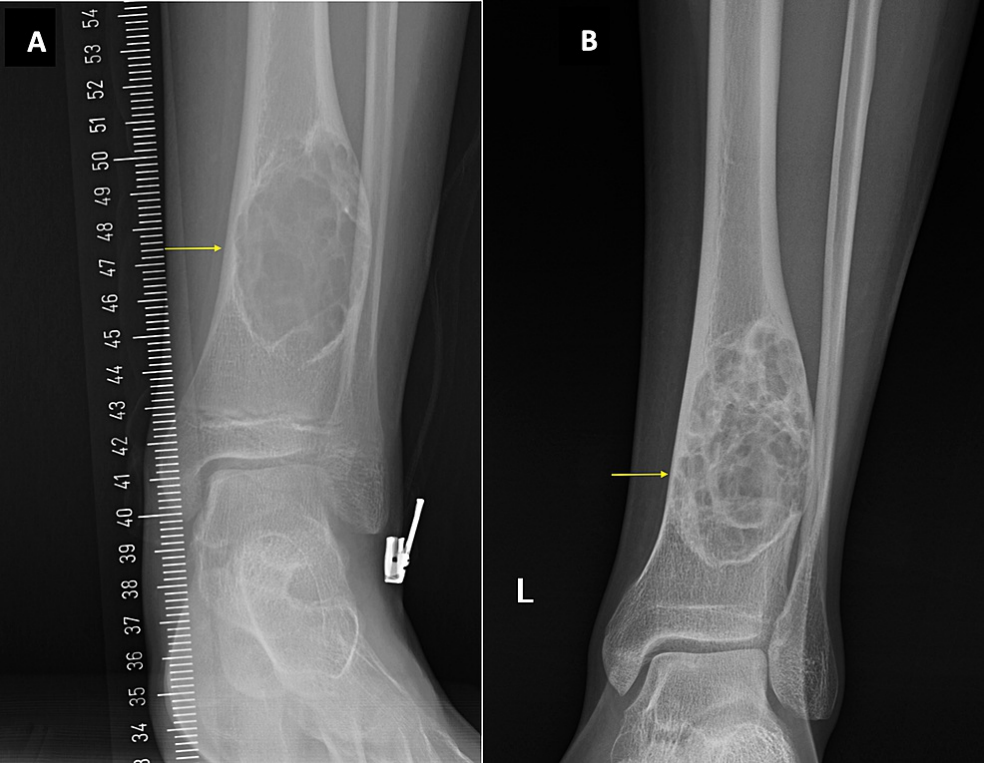
Plain radiographs of Case 2. In Figure 2A, a lucent lesion in the left distal tibia with polycystic borders in keeping with a Ritschl stage B NOF is seen. Figure 2B shows the same lesion three years later. NOF: Non-ossifying fibroma.

Case 2:

A 15-year-old boy presented with a two-month history of left ankle pain possibly related to a sports injury. The ankle was tender to touch on the anterolateral aspect. The foot was externally rotated, and an antalgic gait was noted. A palpable lesion was found on the lateral aspect of the ankle. On imaging, a lesion of 6.7 cm in length was found in the distal diaphysis of the left tibia, a few centimeters above the physis. The lesion occupied the entire width of the diaphysis and was multilobulated with a sclerotic margin, lying against the cortex (Figure 2A). No fracture was noted at the time of presentation. After three years, the NOF was noted to be the same size and in the same location with greater ossifications of the margin (Figure 2B).

**Figure 3:**
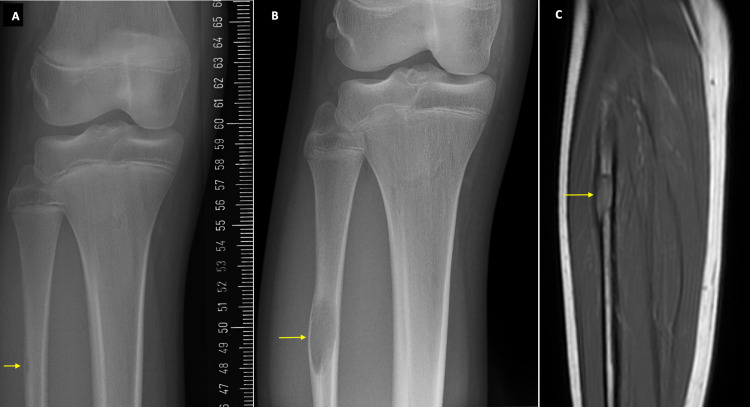
X-ray and MRI images of Case 3. Figure 3A shows a small lucent lesion in the proximal fibula. Figure 3B shows the growth of the lesion seven months later. This lucent lesion is in keeping with a Ritschl stage A NOF. The MRI with contrast in Figure 3C shows the same lesion as in Figure 3B encroaching the medullary cavity. NOF: Non-ossifying fibroma.

Case 3:

An 11-year-old girl came to the ED with acute onset right anterior knee pain after tripping over while on a walk. A few months later, she was seen in the clinic for recurrent falls and knocking knees. Radiographs showed mild genu valgum. A very small translucent lesion was seen in the lateral cortex of the proximal fibula (Figure 3A). A year later, the genu valgum had improved but the patient complained of pain over the tibial tuberosity and mid patella tendon. On re-imaging, radiographs now showed the same translucent lesion but larger in size and in the same location (Figure 3B). The growth of this lesion warranted a T1-weighted MRI which measured a 10 mm x 8 mm x 30 mm lesion (Figure 3C). With gadolinium contrast, peripheral enhancement was noted with marked thinning of the lateral cortex. The lesion was subcortical and encroached on the medullary cavity. There was minimal surrounding edema without periosteal reaction or cortical destruction. Six months later, the NOF was unchanged and no other abnormalities were noted.

**Figure 4:**
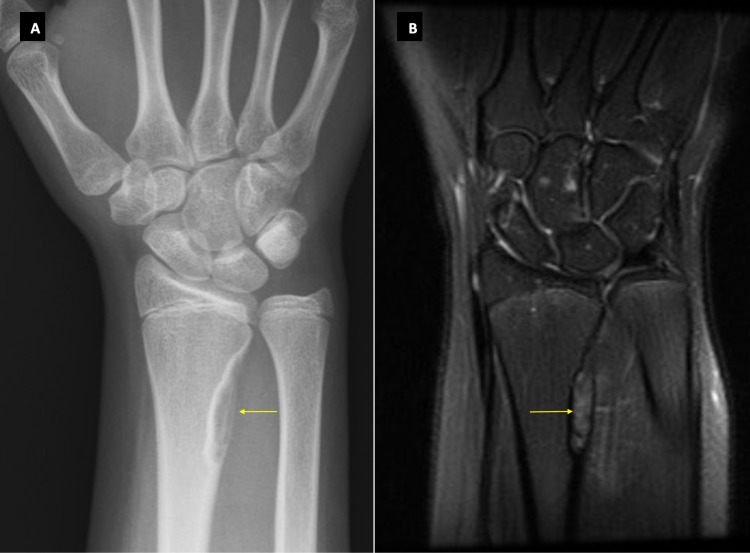
X-ray and MRI images of Case 4. Figure 4A shows a Ritschl stage C NOF with partial calcification from the margins inwards. The same lesion is noted on MRI imaging in Figure 4B. NOF: Non-ossifying fibroma.

Case 4: 

A 14-year-old girl presented with a red and swollen right distal radius and reduced range of movements after falling from a curb onto an outstretched hand. A plain radiograph of the wrist was ordered to check for any fractures. Incidentally, a well-circumscribed cortically based lucent lesion at the lateral aspect of the right distal radial metaphysis was noted in the absence of a fracture (Figure 4A). An MRI was subsequently performed to investigate this lesion further and confirmed a 5 mm x 8 mm x 27 mm non-aggressive lesion of low T1 and high T2 signal, strongly in keeping with an NOF (Figure 4B). A tiny ganglion was also noted near the palmar aspect of the scapholunate ligament. The patient was discharged to the care of her primary care physician.

**Figure 5:**
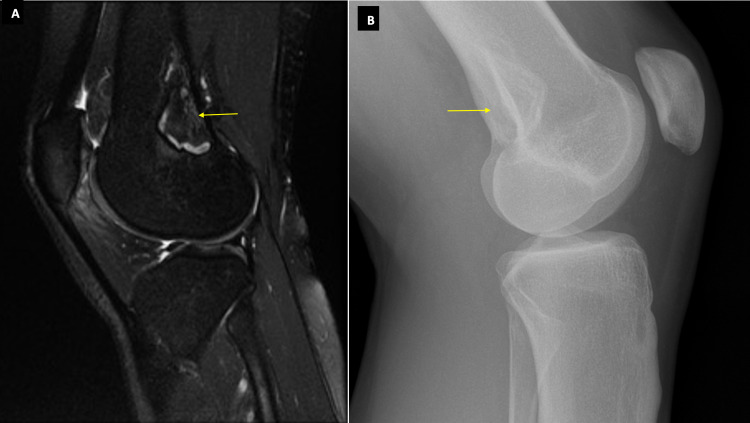
MRI and X-ray images of Case 5. Figure 5A shows a sagittal T1-weighted MRI of the knee showing an NOF. Figure 5B shows a plain lateral-view radiograph of the right knee taken four months later. A sclerotic border with signs of calcification from the margins inwards is seen which is in keeping with a stage C NOF. NOF: Non-ossifying fibroma.

Case 5:

A 27-year-old female dance teacher was referred by physiotherapists to orthopedics with bilateral knee pain which was worse on climbing stairs and dancing. MRI found edema-like signal changes in Hoffa’s fat pad consistent with impingement and suggestive of patellar maltracking. In addition, a 40 mm x 19 mm x 19 mm cortical lesion with sclerotic margins and normal marrow fat was noted in the posterior aspect of the lateral femoral metaphysis, highly suggestive of a healing NOF (Figure 5A). She was told to continue physiotherapy and a follow-up plain film was arranged for four months later (Figure 5B).

## Discussion

On X-ray imaging, NOFs most commonly appear as a well-defined lesion with an eccentric location in the metaphysis of long bones. Due to mild bone remodeling, the cortex over the lesion may appear expanded and thinned [5]. These lesions are not associated with cortical breaches or soft tissue extension. General imaging differentials include aneurysmal bone cyst and chondromyxoid fibroma which warrant further investigation. The distinction between an NOF and its differentials can be achieved based on the patient's history. NOFs often present asymptomatically while more sinister bone lesions present more commonly with pain, swelling, and tenderness. Imaging requests for NOFs should thus be accompanied with relevant clinical information to aid diagnosis and avoid further unnecessary imaging such as CT scans which pose additional high radiation exposure for young patients. 

All patients in our case series presented with NOFs which were asymptomatic and unrelated to their initial presentation. Their radiographic images were all typical of NOFs showing cortically based, non-aggressive lesions with a narrow zone of transition and sharp well-circumscribed borders indicating poor biological activity. Our findings reaffirm the current literature that NOFs can be confidently identified based on clinical presentation and plain radiographs. However, our case series also highlights the high tendency for further MRI investigation to be requested, as seen in three out of four of our patients who had initial plain radiographs. MRIs should rarely be necessary and used only for cases with diagnostic challenges, when lesions are atypically large or at high-risk locations such as the growth plates. 

Herget et al. reported that stage B lesions are at a higher risk of fracture and recommended clinical surveillance at 6-12 month intervals until stage C is reached [2]. However, other variables including NOF size and location have been investigated to identify their contribution to the risk of pathological fracture. It has been reported that NOFs involving more than 50% of the transverse bone diameter have higher risks of pathological fractures [6]. Conversely, there is also evidence showing that the majority of NOFs exceeding these thresholds do not fracture [7]. Sakamoto et al. showed that lower extremity NOFs have less risk of pathological fractures compared to upper limb NOFs [8]. However, the distal tibial NOFs have also been reported to have a high risk of fracture [2]. Additionally, females have been shown to have earlier regression of NOFs which is thought to be due to an accelerated rate of skeletal maturation [9]. The lack of a clear consensus has resulted in follow-up arrangements based on the clinicians’ discretion. This is seen in our case series since only three out of five patients had follow-ups ranging from four months to three years. Greater research is required so that we can stratify our patients by their risk of fracture and provide standardized follow-up regimens to lead evidence-based medical care.

## Conclusions

Although NOFs are very common in the pediatric population, there is a lack of awareness for identifying these lesions as well as guidelines regarding their management. Our case series has shown an overuse of MRI involved in NOF diagnosis. There needs to be greater confidence in identifying NOFs by using X-ray imaging in combination with the patient’s clinical presentation. In this way, increasing awareness of NOFs as benign lesions and highlighting their characteristic findings on plain radiographs can reduce the burden on more expensive imaging modalities and result in fewer unnecessary investigations for the patient. Unlike X-ray imaging, MRIs and CT scans require the patient to lay immobile in a closed space for a fixed period of time, which can be difficult for some patients. Furthermore, CT scans result in an unnecessary greater radiation exposure than X-ray imaging and MRIs produce harsh sounds which may not be tolerated well by some patients, especially children. It is important to be aware that NOFs do not require invasive interventions such as biopsy. By correctly recognizing NOFs on radiographs, physicians can ease the anxiety of patients by reassuring them of the benign nature of these lesions and by avoiding less tolerable investigations. 

Conflicting evidence has been shown regarding variables affecting the pathological fracture risk of NOFs. More rigorous studies are required to evaluate these parameters and provide standardised follow-up guidelines to monitor NOF progression and prevent abnormal growth in the pediatric population.
